# Efficient Computation of k-Nearest Neighbour Graphs for Large High-Dimensional Data Sets on GPU Clusters

**DOI:** 10.1371/journal.pone.0074113

**Published:** 2013-09-23

**Authors:** Ali Dashti, Ivan Komarov, Roshan M. D’Souza

**Affiliations:** Department of Mechanical Engineering, Complex Systems Simulation Lab, University of Wisconsin-Milwaukee, Milwaukee, Wisconsin, United States of America; Koc University, Turkey

## Abstract

This paper presents an implementation of the brute-force exact k-Nearest Neighbor Graph (k-NNG) construction for ultra-large high-dimensional data cloud. The proposed method uses Graphics Processing Units (GPUs) and is scalable with multi-levels of parallelism (between nodes of a cluster, between different GPUs on a single node, and within a GPU). The method is applicable to homogeneous computing clusters with a varying number of nodes and GPUs per node. We achieve a 6-fold speedup in data processing as compared with an optimized method running on a cluster of CPUs and bring a hitherto impossible 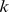-NNG generation for a dataset of twenty million images with 15 k dimensionality into the realm of practical possibility.

## Introduction


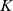
-Nearest neighbor graphs have a variety of applications in bioinformatics [1], [2], data mining [3], machine learning [4], [5], manifold learning [6], clustering analysis [7], and pattern recognition [8]. The main reason behind the popularity of neighborhood graphs lies in their ability to extract underlying information about structure and governing dimensions of data clouds. The 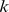-NNG problem is similar to the 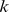-NN problem and a 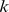-NNG can be built by repeatedly applying the 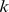-NN query for every object in the input data once a convenient search indexing data structure has been built. Such search data structures include kd-trees [9], BBD-trees [10], random-projection trees (rp-trees) [11], and hashing based on locally sensitive hash [12]. These method focus on optimizing the 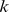-NN search, i.e., finding 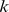-NNs for a set of query points w.r.t. a set of points with which the search data structure is built, ignoring the fact that every query point is also a data point. These methods are generally less efficient compared with one that focuses on 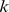-NNG construction directly.

The problem of constructing an exact 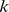-NNGs has been investigated extensively to avoid the 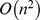 complexity of the brute force method. An 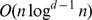 was presented in [13]. [14] presented a 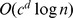 algorithm and [15] presented a worst case 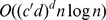. In [16] two practical algorithms for 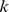-NNGs based on recursive partitioning and pivoting have empirically shown time complexities 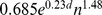 and 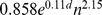, respectively. All these algorithms have a time complexity that is exponential in the dimenision 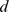 of the data. The same exponential dependence on dimension of time complexity is seen in methods based on space filling curves such as the Hilbert’s curve [17] and Morton’s curve [18]. There are several approximate methods that can handle moderately high dimensional data, typically with a trade-off between speed and accuracy. One set of techniques typically is based on a hybrid of spatial subdivision up to a threshold granularity and small scale brute force evaluation or heuristics for refinement [19]–[21]. These methods rapidly lose accuracy or speed or both when the dimension of the data exceeds 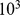.

The curse of dimensionality leads to a belief supported by many researchers that the most efficient method for finding 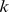-NNGs for high-dimensional data clouds is in fact the brute force method [22]. The brute force algorithm breaks into two parts: distance calculation and comparison. In the distance calculation part, all distances between all points for graph construction are computed. This results in a 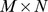 distance matrix, where 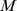 is the number of query points and 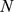 the number of data-base points. Next, each row of the matrix is sorted to get the nearest 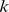 neighbors to each of the query points. Recently, there have been several methods that accelerate brute force 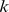-NN and 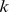-NNGs on graphics processing units. They primarily differ in the manner of selecting 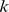 smallest elements in every row in the distance matrix. In [23], [24] each row of the distance matrix is processed by one thread. Each thread uses an modified insertion sort algorithm to select the 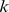 nearest elements. The number of steps that each thread takes to process a given stage is not pre-determined. This can lead to branch divergence and loss of efficiency. Also, since the insertion sort data structure is stored in global memory, this can cause a significant loss in performance due to un-coalesced memory writes. In [25], every row of the distance matrix is processed by a thread block. A heap (one per row) maintains the nearest 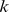 smallest distances. Each thread in the thread block strides through the row reading and storing the element in a buffer if it is smaller than the largest element in the heap. When the buffer fills up, all threads synchronize and then push their elements on to the heap in a serialized manner. The last stage of this algorithm can be extremely expensive especially for large 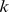. In [26], a thread block is used to process a single row. Each thread in the thread block strides through the array storing the 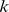 smallest elements in a local heap maintained in global memory. Next, all the thread heaps are merged into 32 heaps in shared memory threads in a single thread warp. Finally a single thread merges the 32 heaps in shared memory to find the 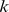 nearest neighbors. Storing heaps in global memory has the same disadvantages of thread divergence and un-coalesced memory write as in [25]. Finally, for 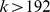 it may not even be possible to execute the method in share memory even on the latest GPUs. All these methods dramatically lose performance for large 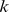. In [27] a radix sort based approach is used to select the 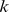 nearest neigbhbors. They claim that for large data sets, especially for a large number of queries, the selection process dominates. A simple complexity analysis suggests that this is quite impossible (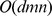 for distance calculation vs 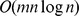 for sorting where 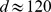 is the data dimension, 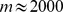 is the number of query points and 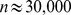 is the number of data points). A closer examination shows that they process each row of the distance matrix in a separate sort. For 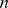 that fit into GPU memory, this process underuses GPU resources.

Our target application is Manifold Embedding to recover structure and conformations from a large data set of images with high noise [28]. The underlying assumption behind manifold embedding states that a cloud of high dimensional data makes a low dimensional hyper-surface in high dimensional space. The generated hyper surface, called a manifold, contains the information about the individual objects, 3D structure of images, and the system that generated the data. The main computational part of manifold embedding is the construction of a 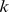-NNG for the image data set that can be normalized to a diffusion map in subsequent stages of algorithm. There are upwards of 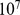 images with each image having 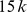 pixels. We require 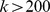 for accurate results. The nature of the data, coupled with the accuracy requirements, makes the brute-force algorithm the only viable alternative. In this paper we describe our parallelized brute-force 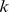-NNG algorithm on a cluster of graphics processing units. We describe three levels of parallel distribution of tasks and data partitioning: between nodes, between multiple GPUs within a node, and finally within the GPU. We have also developed a novel algorithm based on sorting for the comparison and selection step of the brute force 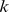-NNG routine. Our benchmarks show that our routine outperforms the best GPU-based comparison and selection methods. Overall, we achieve a 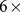 gain in performance over a distributed solution on CPU clusters using the fastest libraries that are available. This bring a hitherto impossible 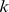-NNG generation for a dataset of twenty million images with 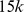 dimensionality into the realm of practical possibility.

## Methods

Given a set of vectors 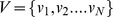 with 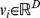, k-NNG finds for each vector 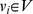 a subset of 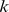 nearest vectors 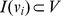. Proximity is defined using a metric 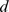. In this work, we are primarily concerned with the Euclidian distance metric given by:
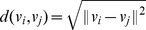



The square of the distance metric can be written as:
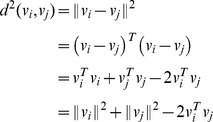



Note that 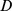 dimensional vector 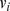 is represented as
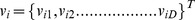



Defining matrices 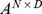, 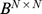 be given by
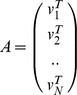


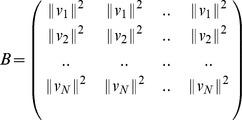



Defining the matrix of squared distances 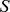 as
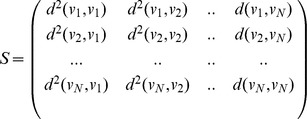



We can now write the following equation
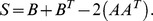



**Figure 1:**
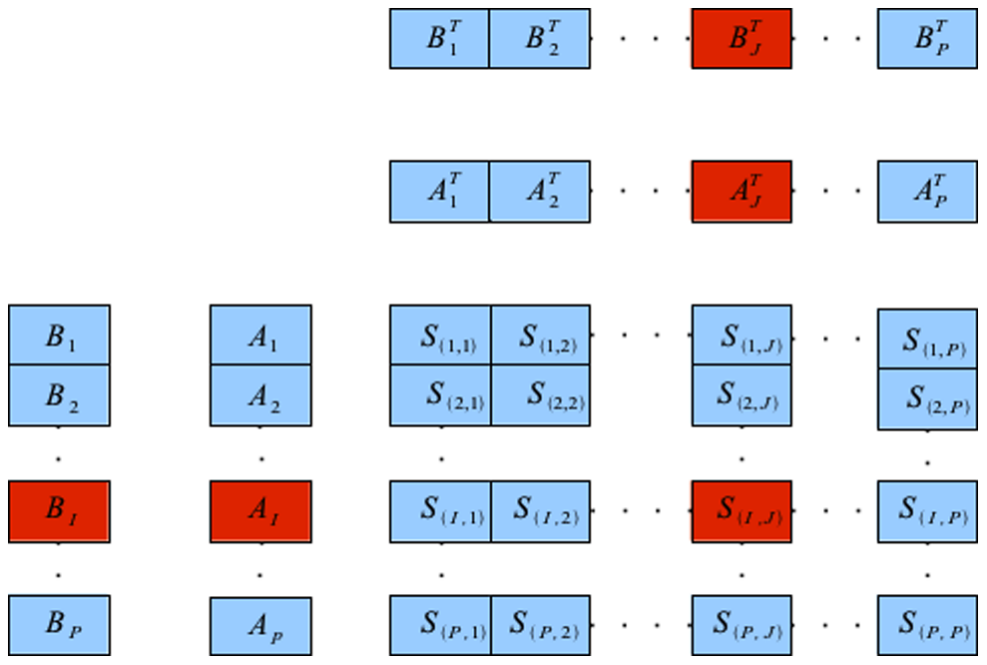
Data partitioning for distributed parallel execution. The squared distance matrix 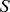 is split into 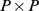 partitions where 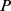 is an integral multiple of 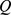, the number of nodes. The computations of the partitions and the subsequent computations of the 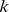-NNs are distributed between different nodes.

Finding the set of 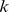 nearest neighbors for vector 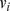 involves sorting the 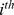 row of 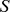 and picking the column indices corresponding to the 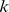 smallest distances. In our application of interest, 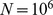 to 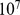. Therefore, computing and storing 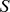 will require 1.8–18 terabytes of memory. Therefore, our approach is to compute 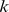 nearest neighbors in parts. As illustrated in Figure 1., the computation of the squared distance matrix 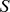 is split into 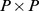 partitions. Consequently, each portion 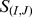 is computed as:
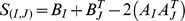
where 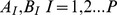 are partitions of 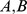 respectively.

Other metrics such as Cosine and Pearson distances can also be incorporated in the 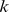-NNG algorithm. The Cosine distance is given by:
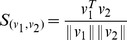



The Pearson distance is given by:
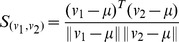
where 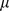 is the average vector of the dataset. The Pearson distance is essentially a centered Cosine distance and therefore an additional pre-processing step for centering is necessary. Clearly, these distance metrics can be formulated in terms of matrix multiplication 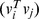 and operations using vector norms. Therefore, the same decomposition and task partitioning applicable in Euclidian case can be used.

### Distribution of Data and Tasks between Computing Nodes

Computing clusters typically have several nodes that are connected by high-speed interconnects. One of the nodes is designated as the head node. The head node typically co-ordinates the tasks between different worker nodes. Each node has its own hard disk. In addition, there is a large shared disk accessible through parallel (I/O) by all nodes that typically hold input data and results. The worker nodes, with smaller local disk space, copy input data as and when required from the shared disk.

**Figure 2:**
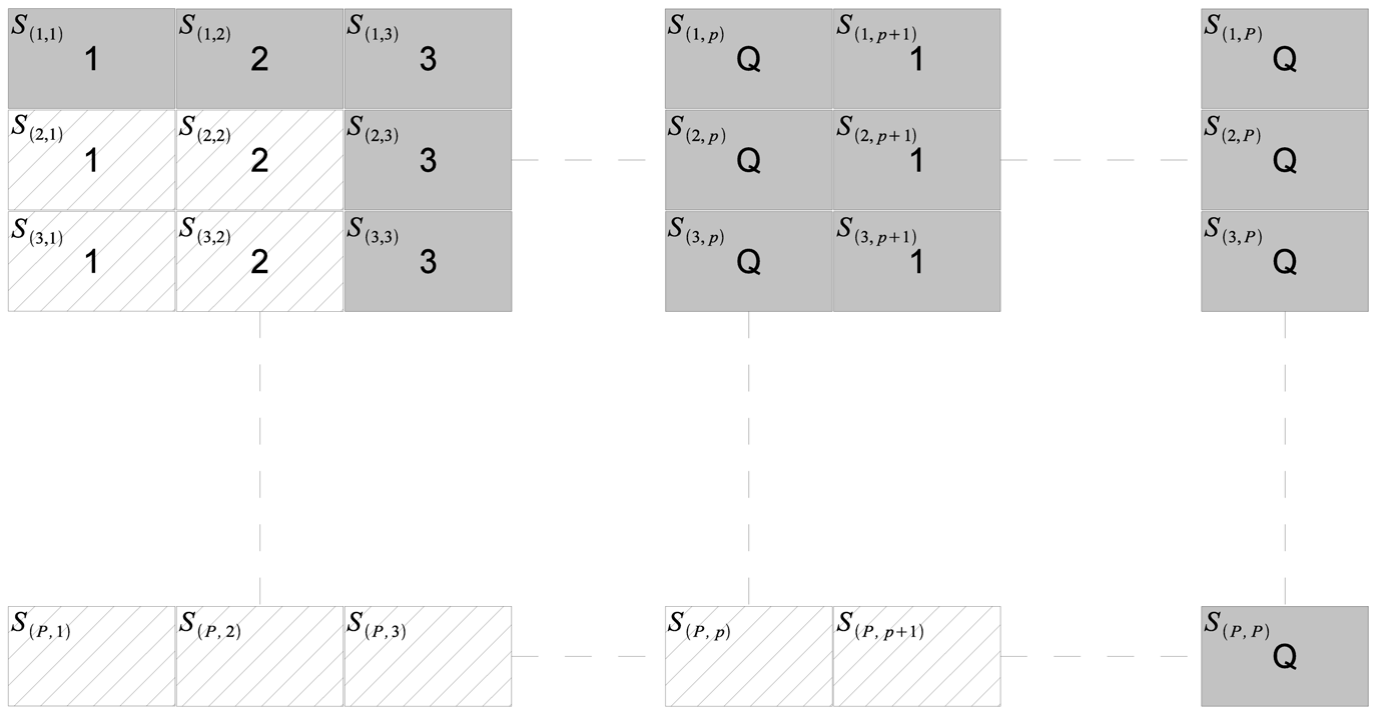
Node assignments for processing partitions of 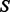. Due to symmetry, 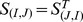. Therefore, only 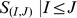 have to be computed. 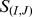 is processed by node 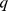 where 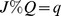.

Table 1 illustrates the steps involved in computing the 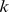-NNG. For load balancing, we distribute computing of the partitions of 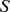 in a block cyclic manner. Since 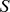 is symmetric, we only need to compute the upper triangular portion, i.e., all sub-matrices 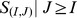. Figure 2 illustrates the task partitioning between different nodes. For example, in block row 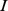, node q will process all sub-matrices 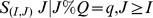. In the beginning, the input data matrix 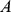 is split into 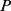 parts. Each node 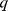 then reads portion 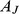 where 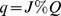 into its local drive. The calculation of the distance sub-matrix 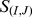 and the calculation of associated 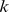-NNs is handled by node 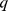, where 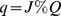. The computation proceeds in a block-row by block-row manner (lines 5–20 in Table 1). Any sub-matrix 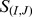 requires inputs 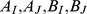. Of these 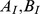 are used by all nodes that are processing the 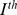 block row. Each node 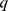 also requires 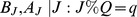. For each block row 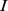, the partition 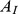 is read in parts in parallel by all nodes from the shared drive. All nodes then share the parts that they each read through an ‘all_gather’ operation [29] to build a local copy of 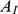 in memory. Each node 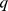 then reads 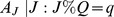 from its local drive. Since the local disk read operation is slower, we use a memory buffer and overlap computation and disk read operation to completely hide latency. We do not actually build the matrix 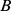. Instead, the vector 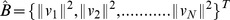 is computed in advance and stored in the RAM of each node. Even for 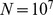 the size of 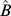 (∼10 MB) is quite small compared to the RAM in each node (48 GB). Each node 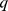 computes the vector norms for all vectors in the partitions 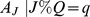 resident on its disk space locally. Using the ‘all_gather’ operation, the nodes can then share data among themselves and build a complete local copy of 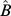.

**Table 1:** Parallel 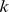-NNG algorithm.

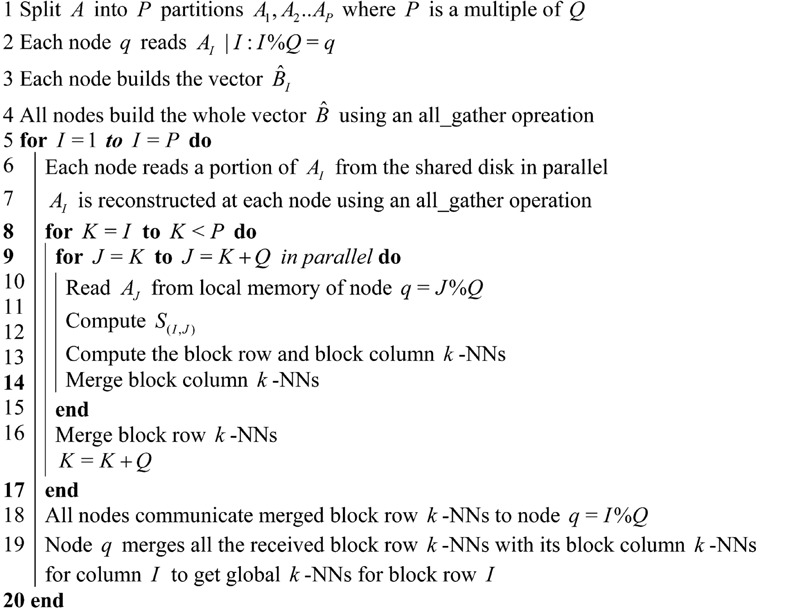

**Figure 3:**
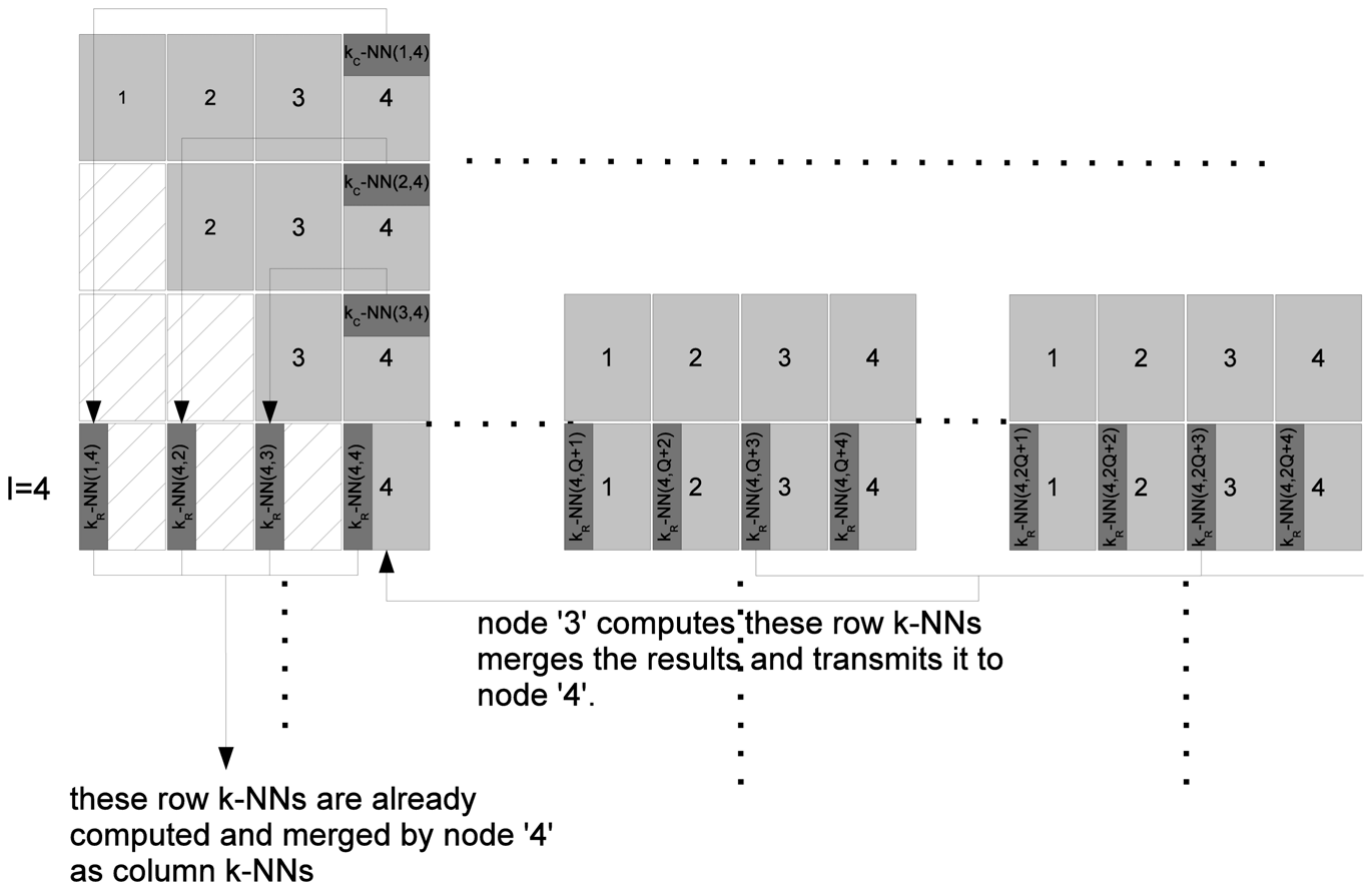
Processing global 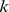-NNs. In this figure node 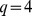 is responsible for calculating the global 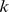-NNs of all vectors that are in 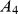. This is done by computing and merging local row 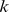-NNs of 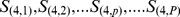. Note that the local row 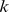-NNs w.r.t. 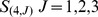 have already been calculated when node 4 calculated local block-column 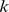-NNs w.r.t. 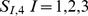. The merged results are stored in a heap. The 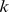-NNs w.r.t 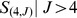 are cooperatively computed by all nodes. For example, node 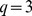 successively computes and merges 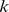-NNs w.r.t. 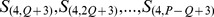. It then transmits the results to node 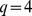, which receives results from other nodes and does a global merge.

Once the partition 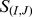 is computed, the local block 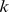-NNs with respect to both the rows (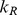-NNs) and columns (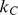-NNs) are computed (lines 9–14 in Table 1). Since the matrix 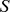 is symmetric, the local block 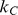-NNs w.r.t. partition 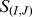 are identical to the local block 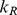-NNs w.r.t. partition 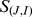. Therefore, each node 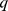 maintains one heap per block column 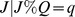 of 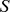 that it processes. Each of these heaps contains the merged local 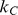-NNs w.r.t. partitions 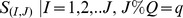. For example, as shown in Figure 3, node 4 maintains one heap for each of the columns 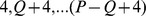. The heap for column 4 will contain the merged block 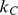-NNs for 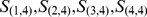. The heap for column 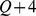 will contain the merged local 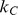-NNs for partitions 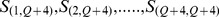.

The node 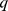 is used to compute the global 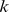-NNs for all vectors in partition 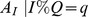. The global 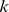-NNs for all the vectors in 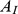 are generated by merging the local block 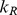-NNs w.r.t. partitions 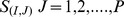. However, the merged results of the local block 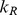-NNs w.r.t all partitions 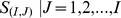 are already available in node 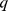 from the local block 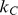-NNs computed when processing rows 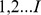. The block 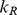-NNs w.r.t. all partitions 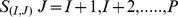 are cooperatively computed by different nodes. Each node maintains a heap to merge the results of finding the local 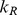-NNs of the partitions that it processes (line 15 in Table 1). At the end, the merged results are communicated to the node processing the global 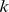-NNs for the block row 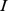 for merging at the global level (line 18 in Table 1). For example, as illustrated in Figure 3, for 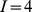, node 3 will compute block 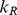-NNs w.r.t. all partitions 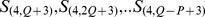. The results will be merged and stored in a heap. At the end of the computation, the results in the heap will be communicated to node 4. Node 4 will have computed all 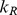-NNs w.r.t. 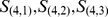 and merged them while processing block rows 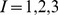. These results and the results communicated to it by all other nodes processing parts of block row 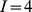 will be merged by node 4 to compute global 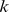-NNs for all vectors in 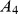.

#### Data partitioning parameters

Our tests reveal that compute time dominates communication time. For a variety of decomposition sizes, we find that communication time is no more than 2% of compute time. Therefore, by making the decompositions as large as possible, any communication costs can easily be masked by overlapping with computing. The size of the partition is therefore governed solely by the amount of node RAM and is given by:
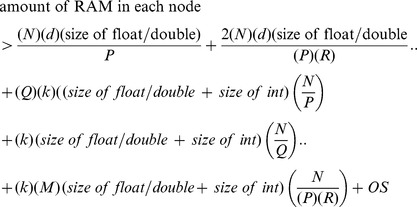
where, 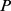 is the smallest multiple of 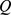 that satisfies the above relation, 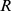 in the size of the partition of 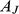 within the node and is dependent on GPU RAM size. Here the first term in the right hand side is the size of 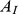, the second term is the size of a buffered portion of 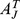 (one buffer portion is for reading from the disk while the second is for feeding the GPUs. These buffers are swapped at the end of computation.), the third term is the size of the buffer to store 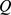 row 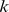-NNs (this data is used to compute the global 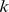-NNs. The data structure is a tuple consisting of a float/double to represent the distance and a int for index.), the fourth term accounts for the block column 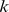-NN storage for all columns being processed by a node, the fifth term represents the memory required for partial column 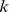-NN generated by the 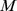 GPUs in each node, and the sixth term represents the memory requirements for the operating system. 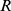 is given by the smallest integer value such that the following relationship holds:
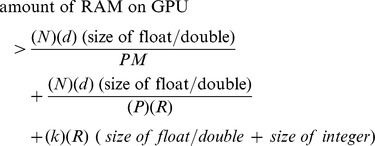
the first term represents the storage for a portion of 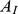, the second term represents the storage for a portion of 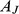 and the third term represents the storage for partial 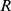 row 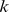-NNs which will be merged by the GPU.

### Distribution of Tasks and Data Within Nodes

We assume that each node has 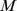 GPUs. In our current setup, 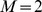. As mentioned previously, each node is responsible for computing a partition 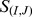 of the squared distance matrix. 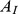
s are read from the head node through parallel I/O. 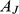
s are read from the local disk. Within the node, 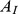 is divided into 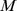 equal partitions. 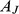 is divided into 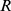 partitions.

**Figure 4:**
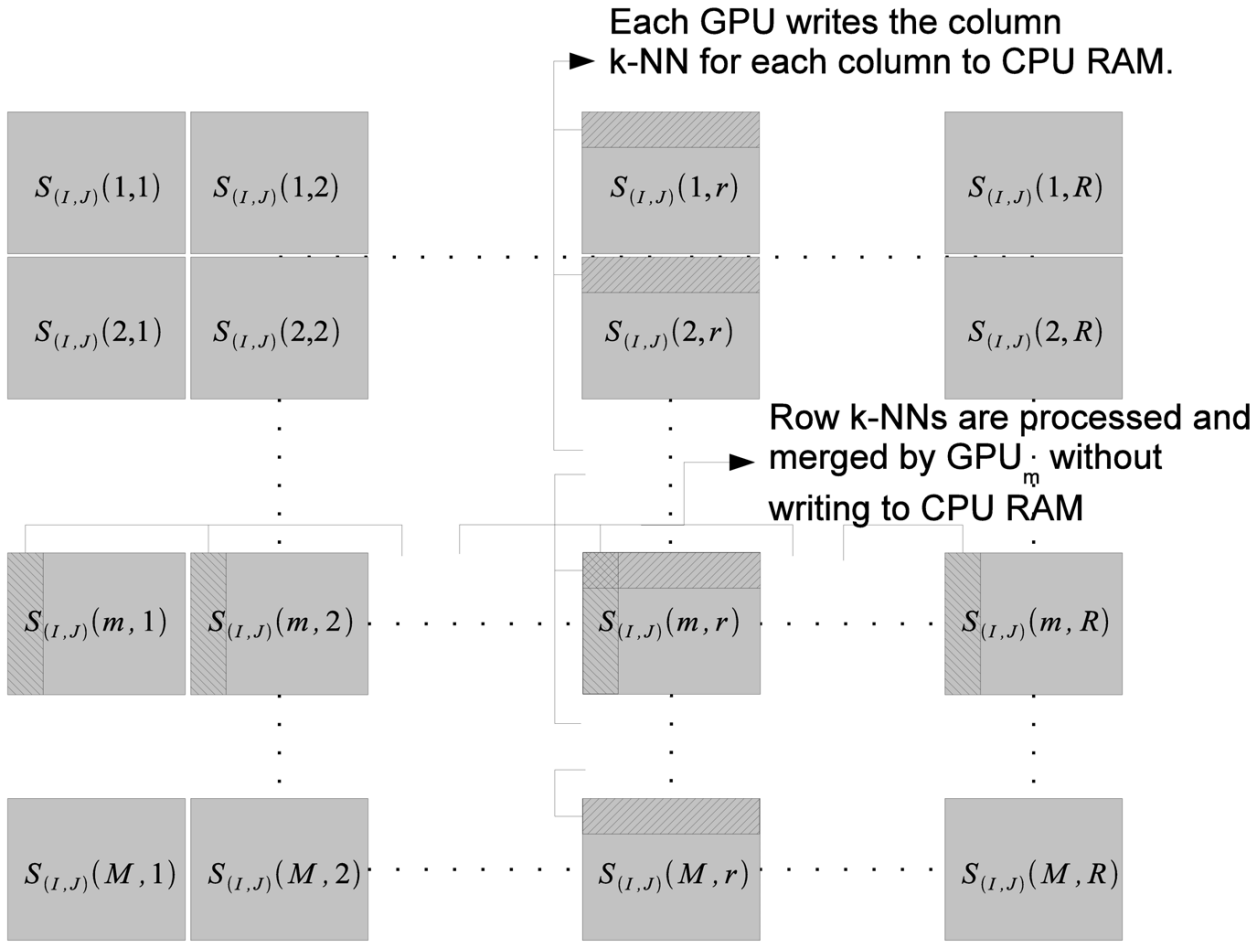
Processing local 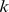-NNs within nodes. The sub-problem assigned to a node is finding the row and column 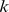-NNs w.r.t 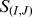. 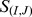 is divided into 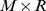 partitions. All partitions 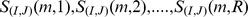 are processed by GPU 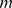. The row 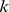-NNs are processed within GPU memory and the merged results are written to CPU RAM. The column 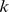-NNs are written to CPU RAM. Later, each of the local column 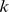-NNs are merged by a single GPU.

Task distribution within each node is described in Figure 4. Each GPU 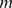 is responsible for computing the sub-matrices 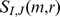 and the associated row and column 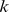-NNs. This process requires portions of the input vector data matrices 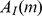 and 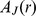. Once again the computation proceeds in a block row manner. Therefore the portion 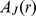 is read by all GPUs while as the portion 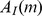 is read by the GPU 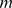. When the computation of 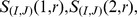 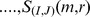 is being done by the 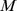 GPUs, the node simultaneously reads the file partition 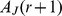 into the RAM. The node also has the norm vector 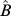 in its RAM. 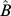 is partitioned in two ways: one has 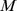 partitions with each of these partitions going to the 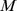 different GPUs and the other has 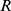 partitions, with each partition being read sequentially by all GPUs. When the computation of 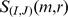 is complete, the column 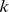-NNs as well as the row 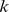-NNs are computed using sorting. The column 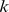-NNs are written back to CPU memory while the row 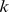-NNs are kept on global memory to be merged. Note that 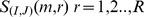 are being processed by the same GPU 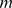, therefore, it makes sense to merge row 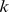-NNs in the GPU memory without write back to CPU RAM. However, 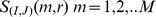 are being processed by different GPUs, therefore, the column 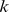-NNs are generated by different GPUs. The accumulated column 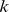-NNs are then merged by one GPU per column. The final result of this operation is the local row and column 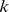-NNs for the partition 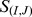.

We use OpenMP multi-threading [30]. Each node runs 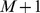 CPU threads. 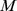 threads control the 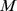 GPUs while one thread is in charge of I/O from the local disk as well as the shared disk.

### Distribution of Tasks and Data within GPUs

The tasks that are accomplished within each GPU include the following:

- Finding vector 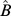 of input data norms
- Dense matrix multiplication to generate the result 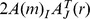

- Summation to find the result 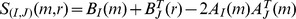

- Finding local 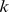-NNs based on 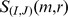

Each of these tasks is coded as *kernels*. In our clusters we have Tesla C2050 compute GPUs from NVIDIA. We use the CUDA programming environment [31] to code our kernels.

#### Finding vector norms

**Figure 5:**
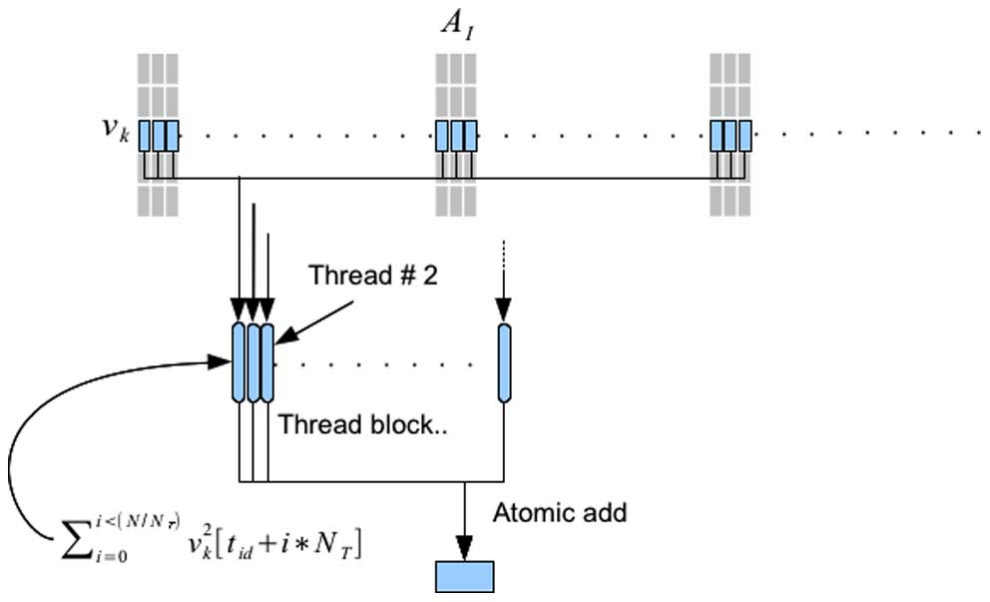
Finding vector norms. Each thread block is assigned to compute the norm of one vector in 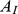. Each thread 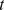 strides through the vector and computes the sum 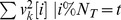, where 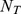 is the number of threads in a thread block. Finally, an atomic add operation is used to add all the sums within each thread into a location in global memory on the GPU.

Finding vector 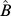 of input data norms is done once in the beginning at the same time that the input files 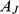 are communicated to each node. For example, if node 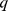 will receive all files 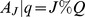. When the file is received, it is partitioned into 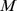 partitions, one partition per GPU. Although there is a library function to calculate vector norms in CUBLAS [32], using it will be inefficient since the vectors are relatively large in number (N = 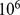 to 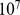) with much smaller dimension 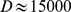. Finding the norm of vectors one at a time will not fully use GPU resources. We have written our own kernel that overcomes underuse of GPU resources by computing multiple vector norms in one kernel invocation.This process is illustrated in Figure 5. Every vector in partition 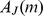 is processed by one thread block. All threads in the thread block cooperatively compute the vector norm. Each thread strides through the vector components adding the square of the entries with a stride length equal to the number of threads in the thread block. Finally, all threads in the thread block write to a single global memory location using atomic-add.

#### Dense matrix multiplication

**Figure 6:**
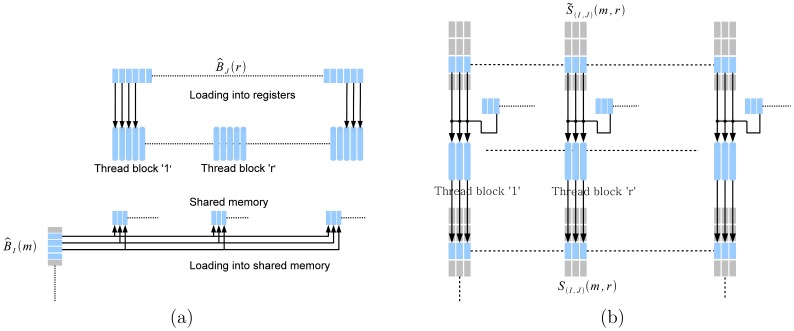
Summation kernel. Calculation of every row of 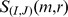 involves 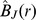 and one element of 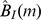 per row. Therefore, each thread loads an element of 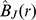 into a register. These data are reused to compute all rows of 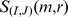. Next, one thread per block reads the corresponding element of 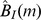 into shared memory. Next, each thread reads an element of 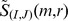 and adds to it the element of 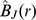, which is in the register and the 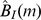 into shared memory to generate the corresponding element of 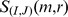.

For dense matrix multiplication 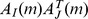, we use the optimized library function from CUBLAS [?]. The result of the dense matrix multiplication 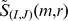 is stored in global memory. For summation, we have written a special kernel to take advantage of the particular structure to minimize memory transactions. Figure 6 illustrates the summation kernel. As mentioned previously, we do not build the matrices 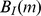 and 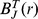 but simply use the portions of the vector of input data norms 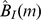 and 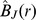. We process 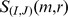 one row at a time. Every thread reads one element of 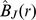 into its register. Next, the thread block reads a section of 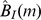 into shared memory. Finally, all threads simultaneously update the entire row of 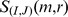. Each thread reads the corresponding element of row 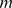 of 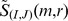, adds to it the corresponding element of 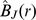 that is in its register, and an element 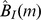 that is in shared memory. In reading an element 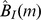 by all threads in the thread block, we use the broadcast mechanism in GPUs.

#### Batch index sort for 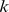-NN

**Figure 7:**
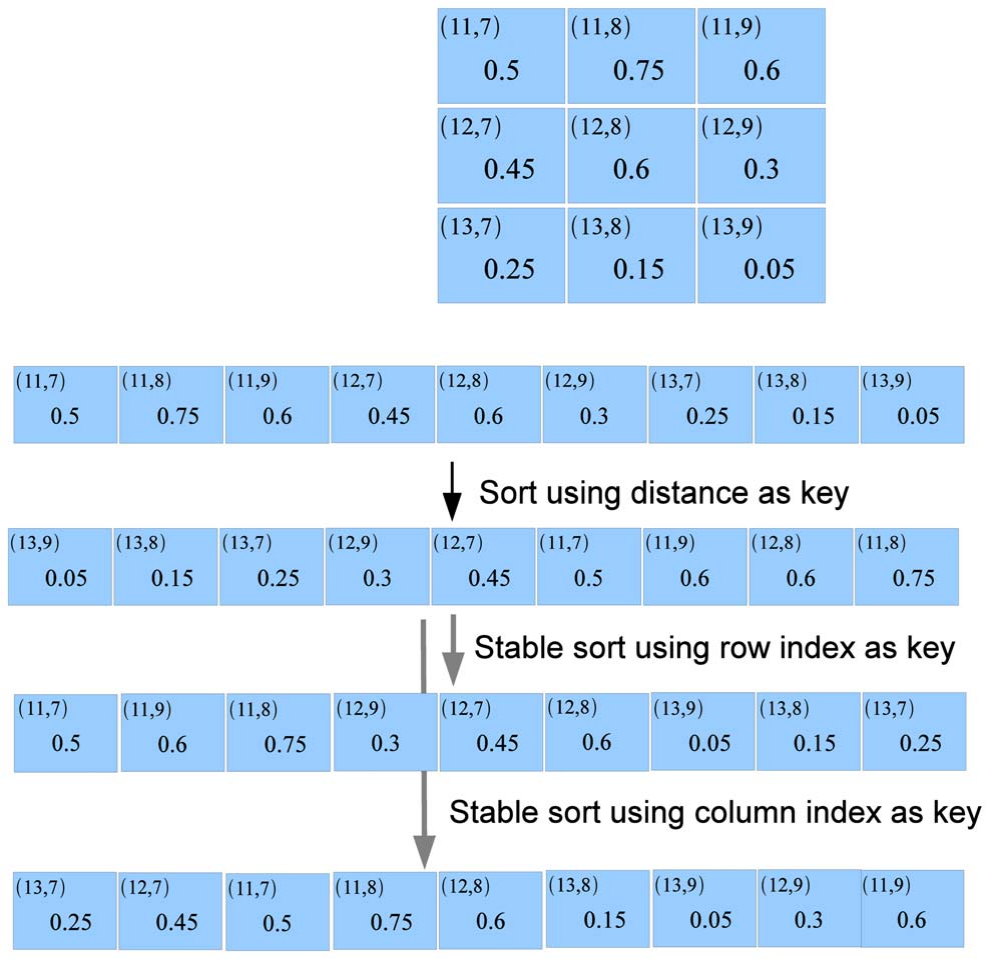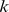-NN using sorting. Here we illustrate the 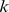-NN search for 3 vectors. The distance matrix is stored along with the column and row indices in a row-major format. First, we sort the entire distance matrix with the distance as the key. The result is next sorted in a stable manner first with the column as the index and then as separately with the row as the index. We then pick the closest 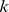 distances both for the columns and the rows results.

We could obviously sort each column and each row separately. However, this is not efficient because the resources on the GPU are not fully used. Also, for sorting according to columns, we will need to execute an expensive operation to rearrange the data in the column major format, Instead, we use a process we called Batch Index Sorting. Note that in GPU RAM, 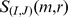 is laid out as a linear array in a row-major format. Each element of 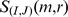 is also then associated with its row index and column index. We use radix sort with the elements of 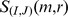 as key. In the next two steps, we execute an order preserving sort of the result of the previous step with the column index as the key. Separately, we also execute an order preserving sort with the result of the first sort with the row index as the key. These two sorting operations generate the nearest neighbors for each column and row. We then execute a separate kernel to extract the 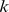-NNs for each row and column w.r.t. 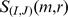. Figure 7 illustrates and example with 3 data vectors and 3 query vectors.

## Results

**Figure 8:**
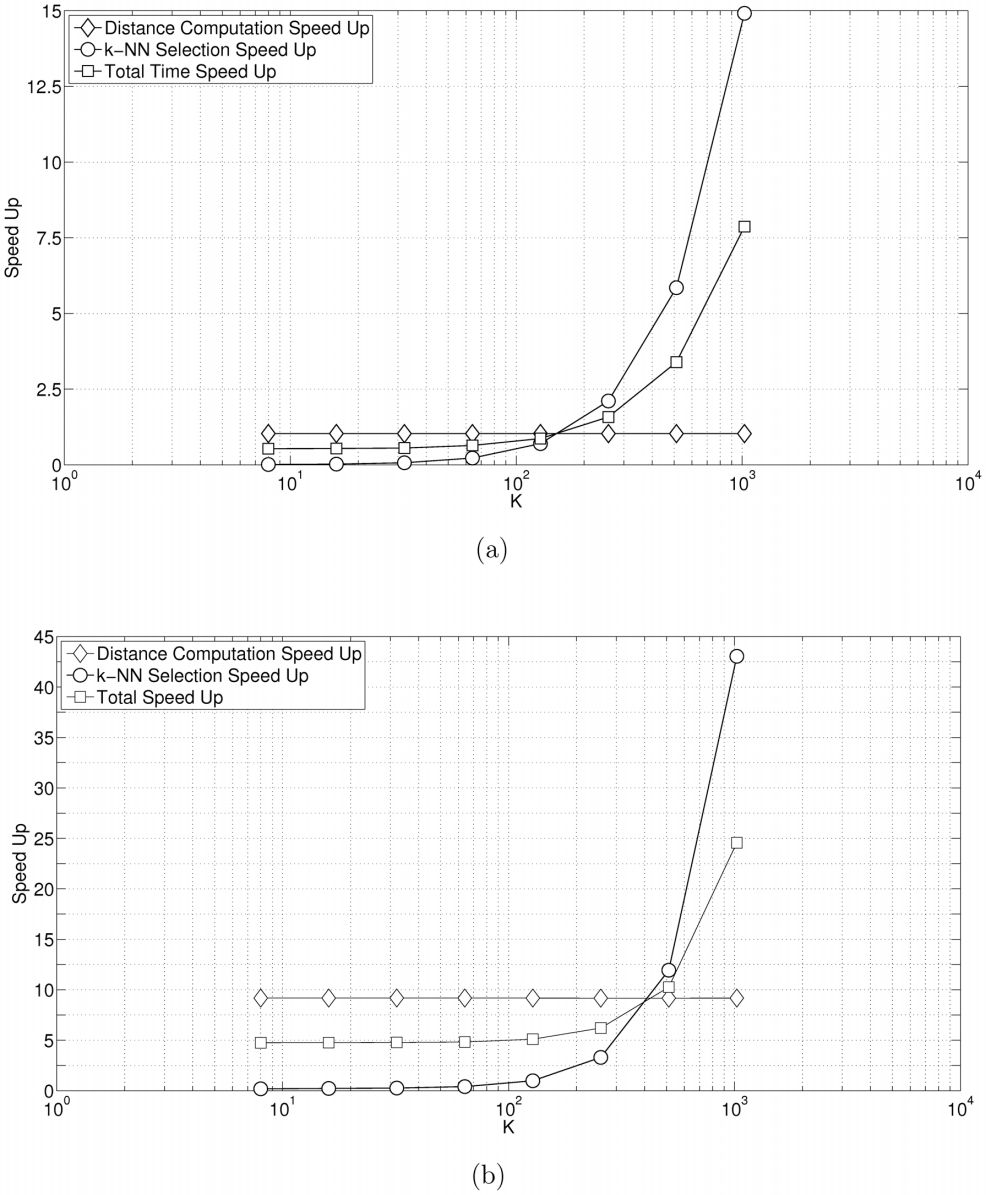
Performance benchmarks for varying 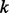. In this test our input data has the dimension 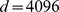 and the number of input objects/vectors 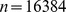. Figure 7(a) shows the performance vs. [23]. Figure 7(b) shows the performance vs. [24].

We tested our implementation against that of [24] and [23]. All implementations were compiled using C++ using appropriate compiler optimization flags. The implementations were run on a NVIDIA Tesla C2050. We used synthetic data sets. We benchmarked distance computation, 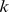-NN selection, and total time. Figure 8 shows the comparison when we varied 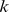. In this test we set the data dimension to 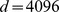 and the number of objects 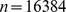. Figure 8(a) shows the speedup versus [23]. The distance computation is almost the same because both works formulate distance computation as a matrix-matrix multiplication and use the optimized CUBLAS library. Our version of 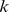-NN selection breaks even with the work of [23] at about 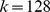 and outperforms it by 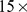 for 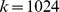. Overall our implementation has a performance advantage of 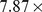. Figure 8(b) shows the speedup versus [24]. For this test we re-formulated the Pearson distance computation to enable the use of optimized matrix-matrix multiplication. Consequently, our distance implementation has a roughly 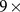 performance advantage. The 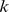-NN implementation is a per thread linear insertion sort with each thread handling one row of the distance matrix. Our implementation breaks even at 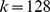 and ends up with a 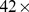 performance advantage when 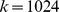. Overall, our implementation has a 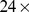 performance gain at 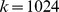.

**Figure 9:**
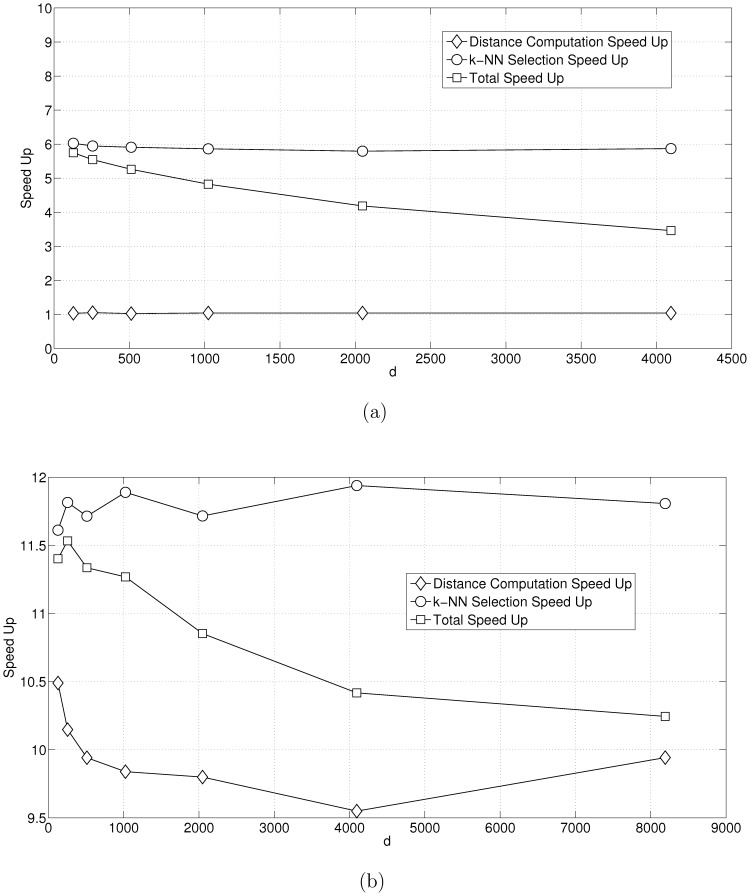
Performance benchmarks for varying 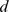. In this test our input data has the number of closest neighbors 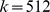 and the number of input objects/vectors 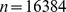. Figure 7(a) shows the performance vs. [23]. Figure 7(b) shows the performance vs. [24].

In the second sets of tests we kept 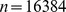 and 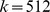 and varied 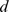. Figure 9(a) shows the comparison with [23]. Both the speedup with respect to distance and with respect to 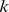-NN selection remains constant at 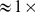 and 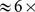, respectively. However, the proportion of time for distance computation increases linearly with 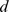. Therefore, we see that the performance gain for total time decreases from 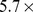 to 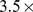. Figure 9(b) shows the speedup with respect to [24]. Once again, the speed up with respect to distance and with respect to 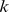-NN selection remains constant at 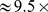 and 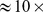, respectively. Once again as 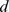 increases, the proportion of time for distance computation increases linearly with 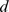. For large 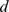, the graph shows stabilization of the overall speedup at 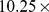.

**Figure 10:**
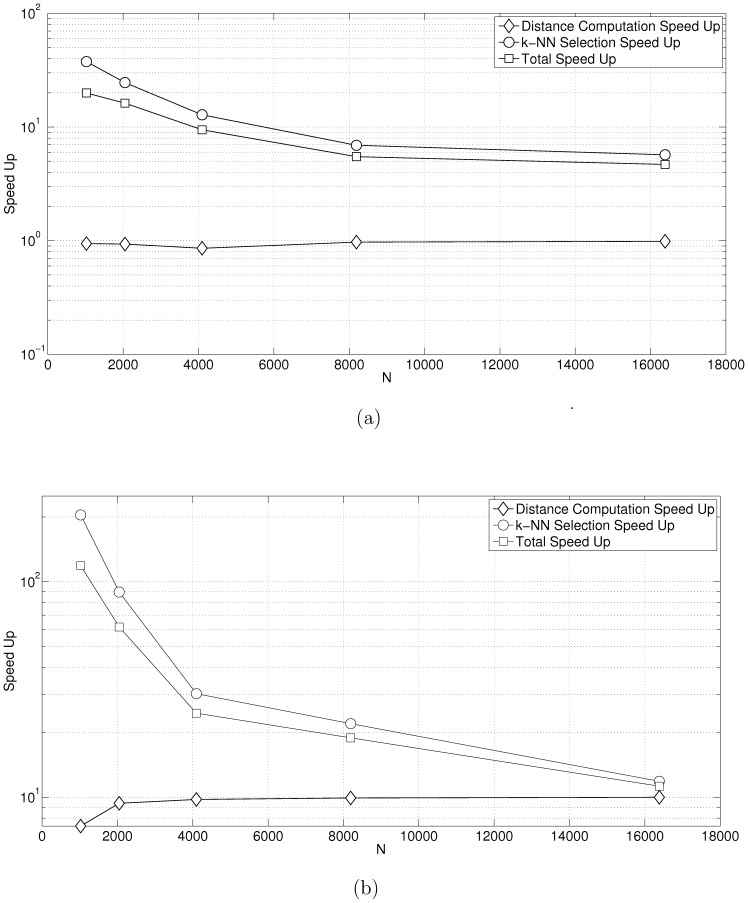
Performance benchmarks for varying 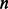. In this test our input data have the dimension 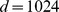 and the number of input objects/vectors 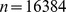. Figure 7(a) shows the performance vs. [23]. Figure 7(b) shows the performance vs. [24]. As 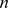 increases, the total performance gain asymptotically approaches that of matrix multiplication because for large 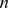, this computation dominates.

In a third sets of tests we kept 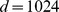 and 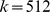 and varied 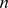. Figure 10(a) shows the comparison with [23]. For small 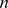, the speedup with respect to selection is 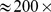. As 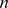 increases, the performance gains taper off to 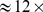. Overall speedup starts off at 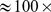 and falls to 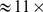. Figure 10(b) shows the comparison with [24]. Once again for small 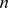, the speedup with respect to selection is 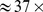. As 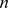 increases, the speedup tapers off to 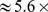. Overall speedup starts off at 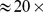 and tapers off to 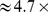. Finally, for the tests with varying 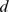 and 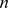, while our implementation was able to handle a model size up to 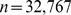, the implementations by [23] could only handle a model size up to 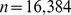.

**Figure 11:**
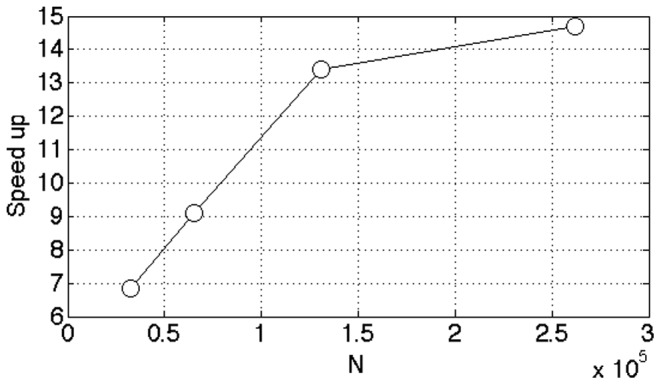
Performance benchmarks for multi-GPU execution. In this test we used 2[24] the 2 GPUs (Tesla 2050) were mounted on a single desktop machine. For our implementation, we use 2 nodes in our GPU cluster and opted to use only one GPU per node. The input data had the dimension 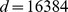, and the number of closest neighbors 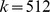.

We also tested our implementation vs. [24] for multi-GPU configuration. While the implementation in [24] requires all GPUs be on a single computer (connected through PCI Express bus with OpenMP multi-threading), our implementation is designed for execution on GPU clusters, i.e., the scalability is much larger. In the tests, we ran the implementation in [24] on two GPUs on a single desktop and our implementation was run on two nodes of a cluster, with each node containing a single GPU. We used a combination of MPI and OpenMP for multi-GPU execution. Figure 11 shows the result. Here 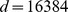 and 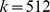. We achieve up to 15× overall speedup. However, for data with a small dimension (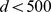) and a small 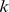 (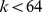), the implementation in [24] can be faster. In fact, for 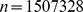, 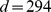 and 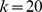, our implementation is roughly 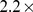 slower. This is mainly because our 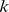-NN algorithm is not as efficient for small *k*s. Our new 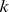-NN algorithm to be published in a subsequent paper is much faster.

**Figure 12:**
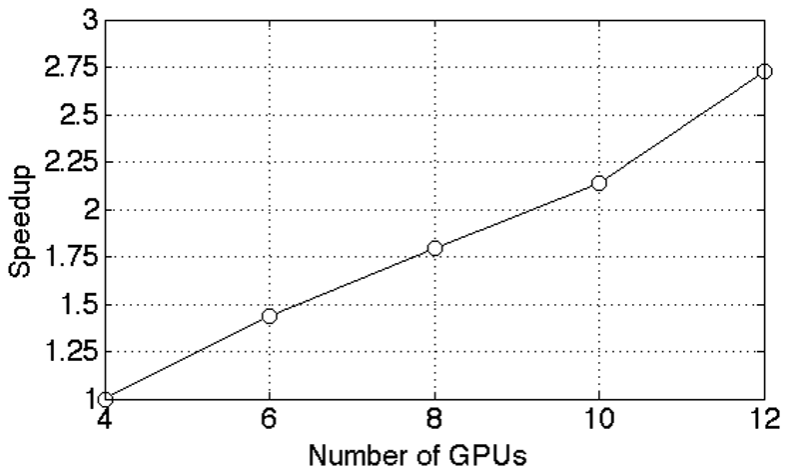
Performance scaling with number of GPUs. In this test we used a data set with 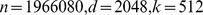. We achieve a performance speedup that is slightly below linear in the number of GPUs.

Figure 12 illustrates the scalability of the method w.r.t. the number of GPUs in the cluster. For this test we used a data set with 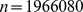, 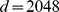 and 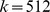. As can be seen from the graph, we achieve a performance gain that is lightly below linear in the number of GPUs. Since each node contains 2 GPUs, we went from using 2 nodes to 6 nodes in the cluster.

To demonstrate the full capabilities of our implementations, we executed 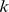-NNG construction for two datasets. The first data set contains two million images of simulated diffraction patterns of a randomly oriented Adenylate kinase (ADK) molecule. Each image has 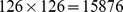 pixels. The second dataset consists of twenty million images of simulated diffraction patterns of melting ADK in ten different molecular conformations. For more information about the structure of data sets, please refer to [28]. Unfortunately, the second data set on our CPU cluster would take approximately 3 months to compute, and therefore, we do not have actual timings.

We evaluated our method with a previous implementation of a neighborhood graph construction using MATLAB technical computing language. The MATLAB implementation took 56 hours on an exclusive CPU cluster with 32 nodes for two million images of diffraction patterns with the use of highly a optimized ATLAS-BLAS library [33] for multi-threaded Matrix-Matrix Multiplication in double precision. The cluster had one Xeon E5420 quad-core CPU per node with 16 kB of L1 cache, 6144 kB or L2 cache and 40 GFLOPS of double precision computing power. Comparison and selection was accomplished using the quick sort algorithm. Since the parallel MATLAB implementation did not take advantage of the symmetry of the distance matrix, one can assume that such an implementation would roughly take about 28 hours. Our GPU cluster had 16 nodes with each node equipped with two NVIDIA Tesla C2050 GPUs. Each Tesla C2050 GPU has a RAM of 3 GB with 506 GFLOPS of double precision computing power. There are 14 multi-processors sharing 720 kB of L2 cache and each multi-processor having 48 kB of user-configurable L1 cache/Share memory. In addition, each of the GPU nodes had two quad-core Xeon E5620. Note that in our GPU cluster, CPUs are used mostly for managing the GPUs and movement of data between nodes and not for computation. Our GPU cluster implementation took 4.23 hours, giving a roughly 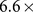 gain in performance.

To investigate the efficiency of our implementation we also benchmarked the most expensive part of the computation, i.e., matrix matrix multiplication, we tested both double and single precision matrix-matrix multiplication on a single GPU (Tesla C2050) vs. a multi-core optimized (with SSE instructions) on a Xeon processor. We achieved a roughly 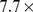 speed-up using GPUs just for matrix multiplication alone. As shown earlier, our complete implementation is slightly worse at a 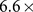 gain in performance. This slight degradation may be attributed to communication overhead while executing on the GPU cluster.

## Discussion

We have successfully implemented a parallel brute-force 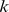-NNG algorithm on a cluster of graphics processing units. We have used multiple levels of parallelism, task distribution, and data partitioning to achieve a roughly 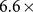 performance gain over an implementation using MATLAB on a comparable CPU cluster. There are several shortcomings in our implementation, as evidenced from comparing the raw float point processing power of the processors. The most expensive part of the brute force 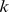-NNG is matrix multiplication. With the best tuned GPU libraries, we see that there is only 50% utilization of GPU resources as opposed to 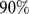 use by finely tuned CPU libraries. A better GPU matrix multiplication library would go a long way toward addressing this deficiency. Another possible route to reducing the computational load is to recursively partition the input data set into sub-divisions with overlaps. This pre-processing step will reduce the computations (distance computation and selection) for each input vector based on set membership. While our current method from comparison and selection is faster than the state-of-the-art, it is clear that by grouping all rows in the distance matrix together and sorting, we are doing a significant amount of redundant work. For example, in sorting distances, there is no need to compare entries in a given row with entries in all other rows to get the smallest 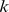 values in the given row. We are currently working on an elegant solution based on quick-sort that will have a complexity of 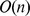. With these improvements we will expect to gain a 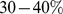 increase in overall performance.
